# Plasma microRNAs are promising novel biomarkers for the early detection of *Toxoplasma gondii* infection

**DOI:** 10.1186/1756-3305-7-433

**Published:** 2014-09-08

**Authors:** Boyin Jia, Zhiguang Chang, Xiaoyan Wei, Huijun Lu, Jigang Yin, Ning Jiang, Qijun Chen

**Affiliations:** Key Laboratory of Zoonosis, Institute of Zoonosis/College of Veterinary Medicine, Jilin University, Xi An Da Lu 5333, Changchun, 130062 China

**Keywords:** *Toxoplasma gondii*, Plasma, MicroRNAs, Biomarker, Real-time PCR

## Abstract

**Background:**

MicroRNAs (miRNAs) have been shown to be present in plasma, which are remarkably stable, and have been suggested as disease biomarkers. *Toxoplasma gondii* (*T. gondii*) is a protozoan parasite that is infective to a wide range of animals and human beings. Previous studies have found that the parasite generated a large number of miRNAs during proliferation and it is known that the spectrum of miRNA expression in the infected hosts is pathogen-specific. To date, there are no reports regarding the application of microRNAs as biomarkers for the early detection of *T. gondii* infection.

**Methods:**

In this study, we investigated the expression patterns of 414 murine miRNAs and tested their expression levels in the plasma after *T. gondii* infection by real-time PCR, with an ultimate purpose of identifying infection-related miRNAs. Three miRNAs in particular, exhibiting prominently elevated expressions, were further validated in a large number of infected mice. The *Toxoplasma* infection-specific miRNAs were confirmed by comparing their expression levels with those of mice infected with *Plasmodium berghei*, *P. yoelii*, *P. chabaudi*, *Cryptosporidium parvum*, Mouse hepatitis virus, and *Staphylococcus aureus*.

**Results:**

Among the 414 miRNA candidates identified by a real-time PCR array, 71 were found to be up-regulated in the plasma of *T. gondii* infected mice. Three of those miRNAs (mmu-miR-712-3p, mmu-miR-511-5p and mmu-miR-217-5p) were prominently expressed in mice infected by both the RH and ME49 strains of *T. gondii*. Additionally, the elevated expression of these miRNAs was *Toxoplasma*-specific.

**Conclusions:**

The levels of the three miRNAs, mmu-miR-712-3p, mmu-miR-511-5p and mmu-miR-217-5p miRNAs, were found specifically up-regulated in plasma of mice after *T. gondii* infection.

## Background

*T. gondii* is an obligate intracellular parasite that causes diverse pathological effects in humans and other warm-blooded vertebrates [1, 2]. Toxoplasmosis, caused by *T. gondii,* is a worldwide parasitic disease that is widespread in Asia, Africa, South America, and Europe [3–5]. The parasite can cause severe disease in immunocompromised patients as well as in pregnant mothers [6, 7]. Toxoplasmosis may cause serious consequences in new-born babies, such as hearing and sight impairment and neurological symptoms [8]. Early treatment of pregnant women could reduce the incidence of sequelae in infected infants [9]. Detection of early-stage toxoplasmosis is a key measure in reducing toxoplasmosis-related health damage.

Serological studies are currently the most common diagnostic method for acute infections; however, they have several disadvantages. Parasite-specific antibodies are not normally present during the early stages of infection, particularly in immunosuppressed patients and in pregnant patients [10, 11]. Other detection methods include PCR assays that are sensitive and fast and can detect parasites from different samples such as amniotic fluid and tissues [12, 13]. However, the detection of *T. gondii* in amniotic fluid remains unsatisfactory because of false negative and false positive findings [14]. Therefore, an ideal method with high specificity and high sensitivity for the early stage diagnosis of *T. gondii* infection is urgently needed.

MicroRNAs (miRNAs) are 21–25 nucleotide noncoding RNA molecules that play important roles in physiological and pathological processes [15]. *T. gondii*-derived miRNAs have been identified in different parasite strains, which will facilitate the dissection of the parasite biology in different biological environments [16, 17]. In addition, the miRNA expression of the host could be affected by the invasion of *T. gondii*[18]. Xiao *et al.* reported that miR-132 was up-regulated in *T. gondii* infected mice and was associated with changes in dopamine receptor signaling [19]. They also found that miR-30c-1, miR-125b-2, miR-23b-27b-24-1 and miR-17 ~ 92 were up-regulated in human macrophages after *T. gondii* infection, which were associated with the anti-apoptosis responses of the host cells [20]. However, the function of both parasite- and host-derived miRNAs in association with parasite infection still needs further study.

Recently, it has been reported that miRNAs are stable enough to be detected in the plasma. In addition, circulating miRNAs from tissues are protected from endogenous RNAse activity [21]. The significance of plasma miRNA levels as biochemical markers for human cancer, such as colorectal cancer, lung cancer, pancreatic cancer, and prostate cancer, has been gradually recognised [22–25]. However, to our knowledge, there are no reports on the characterization of circulating miRNAs in the plasma of patients with *T. gondii* infection.

In this study, a real-time PCR array was applied to measure the levels miRNAs from the plasma of mice infected with *T. gondii*. We focused on the analysis of three miRNAs, mmu-miR-712-3p, mmu-miR-511-5p and mmu-miR-217-5p, which were detected abundantly in mice infected with the RH (Type I) and ME49 (Type II) strains of *T. gondii*. Quantitative analysis of these miRNAs in a large set of plasma samples from mice showed that mmu-miR-712-3p, mmu-miR-511-5p and mmu-miR-217-5p were potentially useful for early stage diagnosis, with a satisfactory degree of sensitivity and specificity.

## Methods

### Plasma sample collections

RH and ME49 strains of *T. gondii* were routinely kept in the laboratory by cell cultivation. Sixty female BALB/c mice (20–25 g) aged 6 to 8 weeks were randomly divided into 3 groups. For plasma collection from mice infected with *T. gondii*, 2 groups of BALB/c mice (20 mice per group) were peritoneally infected with 10^6^ tachyzoites of RH or ME49 strain per mouse. Infection was confirmed by performing Giemsa staining of peritoneal fluid and by PCR [26, 27]. 72 hours after infection, the infected mice were exsanguinated, and the blood was mixed with heparin for the separation of plasma. Blood samples were centrifuged at 1200 g/min for 10 minutes at room temperature to remove the blood cells, followed by a second centrifugation at 12000 g/min for 10 minutes at 4°C to remove cellular components. Plasma samples were stored at −80°C until further processing. Frozen plasma of mice infected with *Plasmodium berghei*, *P. yoelii*, *P. chabaudi*, *Cryptosporidium parvum* (*C. parvum*), Mouse hepatitis virus (MHV), and *Staphylococcus aureus* (*S. aureus*) were also processed as described above. The permission to work with laboratory animals was obtained from the Ethical Committee of the Institute of Zoonosis, Jilin University, China (Permission number 2008-IZ-20).

### RNA isolation

Total plasma RNA was isolated with the mirVana™ miRNA Isolation Kit (Ambion, USA) according to the manufacturer’s protocol. Briefly, synthetic cel-miR-39 was added to each sample as a spike-in control, and total RNA was purified from 400 μl of plasma. RNA was eluted with 100 μl of RNase-free water. The concentration of total RNA samples from plasma was quantified by a Nanodrop 2000 (Nanodrop, USA). The range of the result was from 11.9 to 73.7 ng/μl.

### Real-time quantitative reverse-transcription PCR

Real-time PCR of 414 mice miRNAs was performed. Total RNA from 3 mice infected with the RH strain of *T. gondii* and from 3 healthy controls was used. The reverse-transcription reaction was initiated with 50 ng of total RNA and was carried out in a volume of 20 μl using the miScriptII Reverse Transcription kit (Qiagen, Germany). Cel-miR-39 was used as an internal control. MiRNA concentrations were then confirmed by real-time PCR using the miScript SYBR Green PCR Kit (Qiagen, Germany). Universal primers and the miRNA-specific forward primers were provided by Qiagen. Each reaction was performed in a final volume of 20 μl containing 2 ng of cDNA, 10 μl of 2 × QuantiTect SYBR Green PCR Master Mix, 2 μl of 10 × miScript Universal Primer and 2 μl of 10 × miScript Primer Assay. The ABI Prism 7900 Sequence Detection System (Ambion, USA) was used for amplification and detection. Differences in miRNAs were normalised to cel-miR-39, determined with the ΔCt method, and reported as 2^-ΔCt^.

### Statistical analysis

The quantitative data were analysed with the Mann–Whitney U test. Receiver operating characteristic (ROC) curves were established to examine the accuracy of using miRNAs in early stage detection for diagnosing *T. gondii* infection. A *p* value less than 0.05 was considered statistically significant. All statistical calculations were performed by the SPSS 19.0 software.

## Results

### Preliminary marker selection on a small set of plasma samples

**Figure 1:**
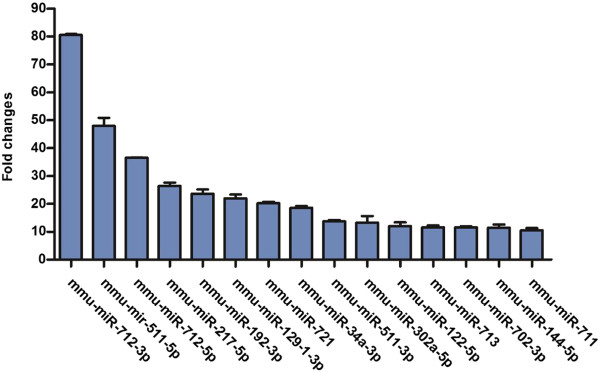
**The top 15 circulating miRNAs with detectable fold changes.** Total RNA extracted from plasma of *T. gondii-*infected mice (n = 3) and healthy controls (n = 3) was analysed by a real-time PCR assay. Y-axis represents the average fold change of the 3 samples.

The aim of this study was to determine whether there are any marked miRNAs with specific expression patterns in mice after a *T. gondii* infection. We performed the initial test using real-time PCR arrays using RNA isolated from *T. gondii*-infected mice and healthy controls. The levels of miRNAs found differed profoundly in the mice before and after infection. In total, 414 miRNAs could be detected by real-time PCR in all 6 samples of the plasma. Of those 414 miRNAs, 71 were significantly up-regulated in the plasma of *T. gondii*-infected mice compared to that of healthy controls. Table 1 shows the 71 miRNAs with their respective detection quantification intensities. Quantification analysis revealed that the top 15 miRNAs differed significantly between the plasma of infected mice and that of normal controls (Figure 1). Of these miRNAs, mmu-miR-712-3p, mmu-miR-511-5p and mmu-miR-217-5p were detected with the highest abundance (the average Ct values were 17.43, 27.45 and 26.08, respectively). Thus, the three miRNAs were chosen for analysis in further studies.

**Table 1:** **Seventy-one differentially expressed (** ***p*** **< 0.05) circulating miRNAs in infected and control plasma samples**

MiRNAs	Fold changes	***p*** -value	Increased or decreased
mmu-miR-712-3p	80.62	9.22E-07	Increased
mmu-mir-511-5p	47.93	0.00133	Increased
mmu-miR-712-5p	36.60	4.99E-06	Increased
mmu-miR-217-5p	26.45	0.00073	Increased
mmu-miR-192-3p	23.69	0.00024	Increased
mmu-miR-129-1-3p	21.99	0.00016	Increased
mmu-miR-721	20.19	0.00018	Increased
mmu-miR-34a-3p	18.54	0.00065	Increased
mmu-miR-511-3p	13.76	0.00024	Increased
mmu-miR-302a-5p	13.26	0.00470	Increased
mmu-miR-122-5p	12.02	0.00291	Increased
mmu-miR-713	11.59	0.00043	Increased
mmu-miR-702-3p	11.59	0.00036	Increased
mmu-miR-144-5p	11.54	0.00285	Increased
mmu-miR-711	10.50	0.00047	Increased
mmu-miR-715	9.47	0.00049	Increased
mmu-miR-532-3p	9.08	0.00070	Increased
mmu-miR-361-5p	9.07	0.00087	Increased
mmu-miR-543-3p	8.78	0.00096	Increased
mmu-mir-194-1-3p	8.75	0.00213	Increased
mmu-miR-362-3p	8.32	0.00267	Increased
mmu-miR-532-5p	8.17	0.00090	Increased
mmu-miR-10a-5p	7.85	0.02967	Increased
mmu-miR-705	7.47	0.00212	Increased
mmu-miR-770-5p	7.31	0.00171	Increased
mmu-miR-218-2-3p	7.27	0.00234	Increased
mmu-miR-493-5p	7.05	0.02691	Increased
mmu-miR-762	7.03	0.00315	Increased
mmu-miR-714	7.01	0.00216	Increased
mmu-miR-187-5p	6.84	0.00173	Increased
mmu-miR-34a-5p	6.67	0.02440	Increased
mmu-miR-744-3p	6.55	0.00688	Increased
mmu-miR-224-3p	6.45	0.01630	Increased
mmu-miR-365-2-5p	6.20	0.00236	Increased
mmu-miR-455-5p	6.09	0.03206	Increased
mmu-miR-122-3p	5.84	0.02323	Increased
mmu-miR-145a-3p	5.82	0.01150	Increased
mmu-miR-599	5.70	0.01257	Increased
mmu-miR-132-3p	5.66	0.03740	Increased
mmu-miR-345-3p	5.61	0.00401	Increased
mmu-miR-320-3p	5.50	0.00795	Increased
mmu-miR-222-5p	5.43	0.00712	Increased
mmu-miR-338-3p	5.39	0.04550	Increased
mmu-miR-323-3p	5.26	0.00462	Increased
mmu-mir-92b-5p	5.21	0.00752	Increased
mmu-miR-760-3p	5.10	0.00402	Increased
mmu-miR-130a-5p	4.94	0.00437	Increased
mmu-miR-212-3p	4.91	0.04220	Increased
mmu-miR-490-5p	4.89	0.00717	Increased
mmu-miR-7a-1-3p	4.73	0.00781	Increased
mmu-miR-363-5p	4.71	0.01730	Increased
mmu-miR-500-3p	4.68	0.04449	Increased
mmu-miR-34b-3p	4.42	0.03696	Increased
mmu-miR-188-3p	4.39	0.01057	Increased
mmu-miR-709	4.34	0.01377	Increased
mmu-miR-30d-3p	4.18	0.02385	Increased
mmu-miR-362-5p	4.09	0.01157	Increased
mmu-miR-448-3p	4.03	0.02179	Increased
mmu-miR-7a-5p	3.94	0.02428	Increased
mmu-miR-196a-5p	3.91	0.04891	Increased
mmu-miR-706	3.89	0.01287	Increased
mmu-miR-361-3p	3.78	0.01351	Increased
mmu-miR-330-5p	3.60	0.02304	Increased
mmu-miR-544-3p	3.20	0.02653	Increased
mmu-miR-135a-1-3p	3.07	0.04930	Increased
mmu-miR-710	2.95	0.04910	Increased
mmu-mir-181a-2-3p	2.82	0.04925	Increased
mmu-miR-302a-3p	0.16	0.01834	decreased
mmu-miR-495-3p	0.16	0.02215	decreased
mmu-miR-454	0.16	0.04909	decreased
mmu-miR-688	0.05	0.02774	decreased

### Independent large-scale validation on plasma samples

**Figure 2:**
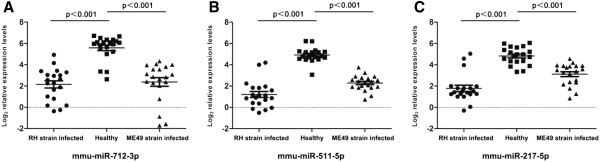
**Validation of mmu-miR-712-3p, mmu-miR-511-5p and mmu-miR-217-5p in plasma samples (n = 60).** Scatter plots of plasma levels of mmu-miR-712-3p **(A)**, mmu-miR-511-5p **(B)**, and mmu-miR-217-5p **(C)** in mice infected with RH and ME49 strains of *T. gondii* (n = 20 each) and in healthy subjects (n = 20). Dark lines represent the mean values where appropriate. Expression levels of the miRNAs (log_2_ scale on the y-axis) were normalised to cel-miR-39.

To further evaluate the expression of mmu-miR-712-3p, mmu-miR-511-5p and mmu-miR-217-5p, 60 plasma samples collected from 20 mice infected with RH strain, 20 mice infected with ME49 strain and 20 healthy controls were investigated by real-time PCR. The expression of these miRNAs was found to be significantly up-regulated in the plasma of RH and ME49 strain infected mice compared to that of the healthy controls by the Mann–Whitney U test (*p* < 0.001, Figure 2). However, no significant changes of the three miRNAs in the plasma of RH strain-infected mice compared to that of ME49 strain-infected mice were observed.

**Figure 3:**
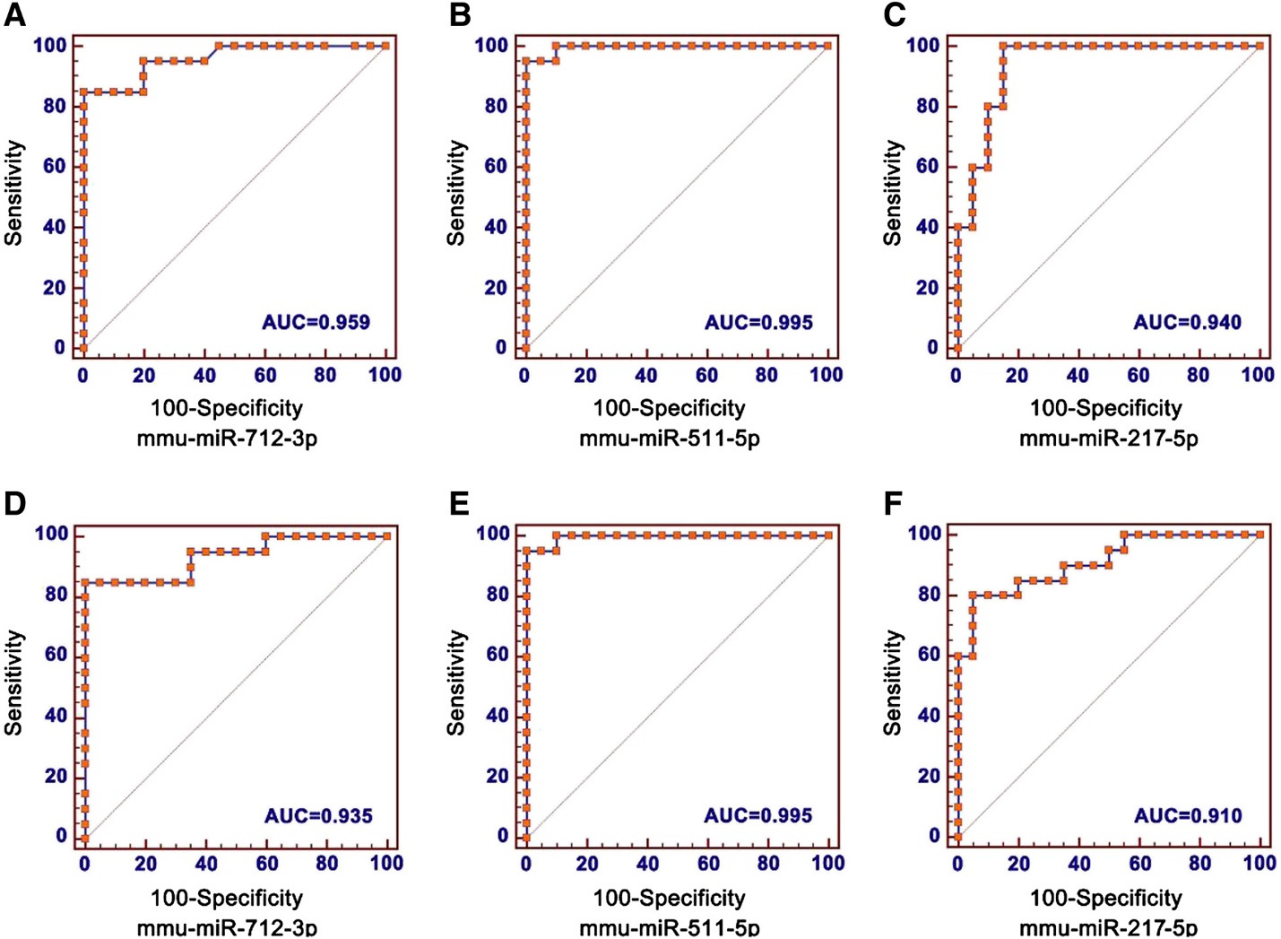
**ROC curve analysis of the expression of plasma levels of mmu-miR-712-3p, mmu-miR-511-5p and mmu-miR-217-5p. A**, **B**, and **C** represent ROC curves of the three miRNAs in mice infected with RH strain of *T. gondii* compared to healthy controls. **D**, **E**, and **F** represent ROC curves of the three miRNAs in mice infected with ME49 strain of *T. gondii* compared to healthy controls. **(A)** Plasma mmu-miR-712-3p yielded a ROC curve value of 0.959 (95% Cl, 0.844 to 0.996), sensitivity of 85%, and specificity of 100%. **(B)** Plasma mmu-miR-511-5p yielded a ROC curve value of 0.995 (95% Cl, 0.902 to 1.000), sensitivity of 95%, and specificity of 100%. **(C)** Plasma mmu-miR-217-5p yielded a ROC curve value of 0.940 (95% Cl, 0.817 to 0.990), sensitivity of 100%, and specificity of 85%. **(D)** Plasma mmu-miR-712-3p yielded a ROC curve value of 0.935 (95% Cl, 0.810 to 0.988), sensitivity of 85%, and specificity of 100%. **(E)** Plasma mmu-miR-511-5p yielded a ROC curve value of 0.995 (95% Cl, 0.902 to 1.000), sensitivity of 95%, and specificity of 100%. **(F)** Plasma mmu-miR-217-5p yielded a ROC curve value of 0.910 (95% Cl, 0.776 to 0.977), sensitivity of 80%, and specificity of 95%.

ROC curve analyses revealed that the plasma levels of mmu-miR-712-3p, mmu-miR-511-5p and mmu-miR-217-5p were also significantly elevated in mice infected with the RH strain of *T. gondii* compared to healthy controls, with ROC curve areas of 0.959 (95% confidence interval, 0.844 to 0.996), 0.995 (95% CI, 0.902 to 1.000) and 0.940 (95% CI, 0.817 to 0.990), respectively (Figure 3A, B and C). The miRNAs were also markedly increased in mice infected with the ME49 strain of *T. gondii* compared to healthy controls with an AUC (area under the ROC curve) of 0.935 (95% CI, 0.810 to 0.988), 0.995 (95% CI, 0.902 to 1.000) and 0.910 (95% CI, 0.776 to 0.977), respectively (Figure 3D, E and F). In mice infected with RH strain and compared to control mice, a cut-off value of 4.94 for mmu-miR-712-3p yields a sensitivity of 85% and a specificity of 100%; at a cut-off value of 4.21 for mmu-miR-511-5p, the optimal sensitivity and specificity were 95% and 100%, respectively; and at a cut-off value of 2.01 for mmu-miR-217-5p, the sensitivity was 100% and the specificity was 85%. Meanwhile, for mice infected with ME49 strain and compared to healthy controls, at a cut-off value of 4.34 for mmu-miR-712-3p, the sensitivity was 85% and the specificity was 100%; at a cut-off value of 3.77 for mmu-miR-511-5p, the optimal sensitivity and specificity were 95% and 100%; respectively; and at a cut-off value of 4.20 for mmu-miR-217-5p, the sensitivity was 80% and the specificity was 95%.

### The elevated expression of miRNAs was specific to *T. gondii*infection

**Figure 4:**
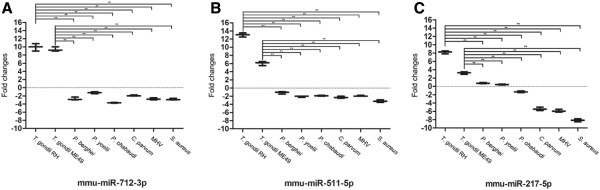
**Relative levels of plasma mmu-miR-712-3p (A), mmu-miR-511-5p (B), and mmu-miR-217-5p (C) in mice infected with** ***T. gondii*** **RH and ME49 strains,** ***P. berghei*** **,** ***P. yoelii*** **,** ***P. chabaudi*** **,** ***C. parvum*** **, MHV, and** ***S. aureus*** **compared to healthy controls.** The levels of the three miRNAs were specifically up-regulated in the plasma of mice infected with *T. gondii* compared to that in the plasma of mice infected by other organisms (**p < 0.001).

We further investigated if the three elevated circulating miRNAs in plasma, mmu-miR-712-3p, mmu-miR-511-5p and mmu-miR-217-5p, were host specific responses to *T. gondii* infection. The expression levels of the three miRNAs in mice infected with *P. berghei*, *P. yoelii*, *P. chabaudi*, *C. parvum*, MHV, and *S. aureus*were compared to the expression levels of mice infected with *T. gondii*. We surprisingly found that the expression of the three miRNAs was up-regulated only in *T. gondii*-infected mice and down-regulated in the mice infected by the other agents (*p* < 0.001, Figure 4).

## Discussion

Accumulated evidence suggests that small molecules in body fluids are novel markers for the clinical diagnosis of diseases [28–30]. Measurement of plasma miRNA concentrations comprises a very promising field for clinical applications. Tumour-derived miRNAs were first shown to be present in the plasma in 2008 [21]. Lawrie *et al.* reported that miRNA-21 was a potentially non-invasive diagnostic marker for B cell lymphoma, which was associated with relapse-free survival [31]. McDonald *et al.* found that miRNA concentrations were higher in the plasma than in sera and that miRNAs were quite stable when frozen for 72 h [32]. During the last decade, circulating miRNAs as stable blood-based biomarkers have been intensively investigated for the early detection of colorectal cancer, lung cancer, pancreatic cancer, and prostate cancer.

To date, the correlation between plasma miRNA levels and *T. gondii* infection has not been investigated. Here, we found that the detection of circulating miRNAs in the plasma of *T. gondii-*infected mice was feasible and that 15 miRNAs were found to be significantly up-regulated in the plasma of *T. gondii*-infected mice compared to healthy mice, with fold changes >10. The expression of the three miRNAs mmu-miR-712-3p, mmu-miR-511-5p and mmu-miR-217-5p were further assessed in a large number of mice infected with either RH or ME49 strain of *T. gondii.* We show that the three miRNAs were consistently up-regulated in the infected mice.

The reliability of the three miRNAs as markers for *T. gondii* infection was further scrutinised by ROC analysis, an analytical method frequently applied in previous studies. The AUC values of the tests of the three miRNAs in the infected mice were between 0.910 and 0.995 with sensitivities between 80% and 95%, and a specificity of 100%. Thus, the three miRNAs are likely to be specific responses to *T. gondii* infection. The miRNAs mmu-miR-712-5p and mmu-miR-712-3p are the mature forms of mmu-miR-712, but the locations of their coding genes are still uncertain and their function unknown. Although both mmu-miR-712-5p and mmu-miR-712-3p were up-regulated in the plasma samples of *T. gondii-*infected mice, mmu-miR-712-3p was more significantly up-regulated. Therefore, we chose mmu-miR-712-3p for further investigation. In previous studies, Son *et al.* reported that mmu-miR-712 derived from pre-ribosomal RNA induced endothelial inflammation and atherosclerosis [33]. Mmu-miR-712-3p may be involved in vascular smooth muscle cell calcification by disrupting Ca^2+^ efflux proteins, which is not related to *T. gondii* infection [34]. Mmu-miR-511-5p was found significantly up-regulated in the brain tissues of *Angiostrongylus cantonensis* (*A. cantonensis*) infected mice. This miRNA might play important role in the regulation of eosinophilic meningitis caused by *A. cantonensis* infection [35]. MiR-511 has been reported to positively regulate TLR4, as well as control macrophage production and activation [36]. Macrophages are important cells involved in the immune response to *T. gondii* infection and have been shown to inhibit parasite invasion and replication [37]. The elevated expression of mmu-miR-511-5p was likely a sign of host immune responses to *T. gondii* infection. However, further experiments are required to reveal the function of mmu-miR-511-5p during *T. gondii* infections. Another over-expressed miRNA from the plasma of mice with *T. gondii* infection is mmu-miR-217-5p, which is known to be encoded by a gene located on chromosome 11. Previous studies indicated that miR-217 may act as a tumour suppressor, as suggested from a study on renal cell carcinoma and pancreatic ductal adenocarcinoma [38, 39]. Up-regulation of miR-217 could decrease the expression of KRAS protein, thereby inhibiting tumour cell growth [39]. However, the function of mmu-miR-217-5p in mice infected with *T. gondii* remains to be investigated.

To further determine that the up-regulation of mmu-miR-712-3p, mmu-miR-511-5p and mmu-miR-217-5p was specific response to *T. gondii* infection, we compared the expression of these three miRNAs in mice infected with *T. gondii* to mice infected with *P. berghei*, *P. yoelii*, *P. chabaudi*, *C. parvum,* MHV, or *S. aureus*. The results showed that these three miRNAs were significantly up-regulated in mice infected with *T. gondii* but down-regulated in mice infected with other pathogens. The three murine *Plasmodium* species, *C. parvum* and *T. gondii* are all members of the phylum Apicomplexa, and they all cause severe infections in mice; however, the miRNA expression responses in the hosts were quite different. Furthermore, the expression of the three miRNAs was also found to be down-regulated in mice infected with MHV and *S. aureus.* Thus, the data collectively suggest that the elevated responses of mmu-miR-712-3p, mmu-miR-511-5p and mmu-miR-217-5p in *T. gondii* infected mice were parasite-specific.

## Conclusion

We report the evidence that three miRNAs, including mmu-miR-712-3p, mmu-miR-511-5p and mmu-miR-217-5p, are significantly up-regulated in the plasma of mice after *T. gondii* infection*,* which may lead to the discovery of novel biomarkers for *T. gondii* infection.
